# Laser-Casein phosphopeptide effect on remineralization of early enamel lesions in primary teeth

**DOI:** 10.4317/jced.52165

**Published:** 2015-04-01

**Authors:** Naser Asl-Aminabadi, Ebrahim Najafpour, Mohammad Samiei, Leila Erfanparast, Somayeh Anoush, Zahra Jamali, Fatemeh Pournaghi-Azar, Sina Ghertasi-Oskouei

**Affiliations:** 1 Professor, Department of Paediatric Dentistry, Faculty of Dentistry, Tabriz University of Medical Science, Tabriz, East Azerbaijan, Iran; 2 Assistant Professor, Department of Paediatric Dentistry, Faculty of Dentistry, Tabriz University of Medical Science, Tabriz, East Azerbaijan, Iran; 3 Assistant Professor, Department of Endodontic, Faculty of Dentistry, Tabriz University of Medical Science, Tabriz, East Azerbaijan, Iran; 4 Post graduate Student, Department of Paediatric Dentistry, Faculty of Dentistry, Tabriz University of Medical Science, Tabriz, East Azerbaijan, Iran; 5 Assistant Professor, Department of Oral Medicine, Faculty of Dentistry, Tabriz University of Medical Science, Tabriz, East Azerbaijan, Iran; 6 Assistant Professor, Department of Operative Dentistry, Faculty of Dentistry, Tabriz University of Medical Science, Tabriz, East Azerbaijan, Iran; 7 Research Assistant, Faculty of Dentistry, Tabriz University of Medical Science, Tabriz, East Azerbaijan, Iran

## Abstract

**Background:**

The aim of this study was to assess the effect of Nd:YAG laser irradiation following casein phosphopeptide-amorphous calcium phosphate (CPP-ACP) application on calcium and phosphate concentration and surface microhardness (SMH) of enamel surface in artificial white spot lesions of primary teeth.

**Material and Methods:**

Eighty teeth with artificial white spot lesions were randomly divided into four groups: (A) distilled and deionized water, (B) Nd:YAG laser, (C) CPP-ACP crème, & (D) CPP-ACP plus laser. SMH was measured using Vickers diamond indenter in Vickers Hardness Number (VHN). Two samples of each group were analyzed using scanning electron microscope (SEM). The results were analyzed with the SPSS 17/win.

**Results:**

The subjects of group D demonstrated a significant increase in the calcium and phosphate contents of enamel surface compared to those of groups A (P < 0.001, P < 0.001), B (P < 0.001, P < 0.001) and C (P = 0.024, P = 0.04), respectively. A statistically significant difference was seen for mean VHN between groups A and B (P = 0.002). SEM evaluations confirmed the results.

**Conclusions:**

The combination of Nd:YAG laser and CPP-ACP crème could be recommended as an effective preventive modality for remineralizing of white spot lesions in primary teeth.

** Key words:**CPP-ACP, enamel remineralization, microhardness, Nd:YAG, primary teeth, SEM.

## Introduction

Dental caries can be prevented when demineralization and remineralization processes are in a balance (1,2). Topical applications of fluoride have been the primary method for stabilization of early carious lesions through remineralization, and thus, fluoride has had a profound effect on reducing caries prevalence. However, dental fluorosis remains an issue of concern, especially in young children. Combinations with other methods and non-fluoride agents have been suggested as alternatives in this regard (3).

An adjunct method in the prevention of caries is the use of laser (4). Laser irradiation causes longitudinal exposure of the enamel rods as a consequence of the micro-explosion, resulting in morphology changes in the enamel through melting and recrystallizing processes, and creation of larger hydroxyapatite crystals on the surface. This makes enamel less permeable to acid penetration and also affects the mineral composition of its deeper layers (5-8). In addition, the heating and melting processes results in significant reduction of carbonate content of hydroxyapatite up to a complete loss resulting in enamel that is more resistant to demineralization (8,9). Laser irradiation in this regard, however, should be applied at a low level of energy to preserve the integrity of tooth enamel (10). To obtain a higher level of resistance to demineralization in enamel, lasers such as Nd:YAG can be combined with fluoride application (11-14).

Casein phosphopeptide-amorphous calcium phosphate (CPP-ACP) is a milk product which has been shown to be effective in the remineralization of incipient enamel lesions through providing a super-saturated saliva with calcium and phosphate reservoir (15-18). In addition, amorphous calcium fluoride phosphate (ACFP) forms as a result of interaction between the CPP-ACP and fluoride ions, and stabilizes by the CPP on the tooth surface. CPP also appears to have an inhibitory effect on the adherence and presence of cariogenic streptococci (19,20). The ecological shift caused by the calcium and phosphate incorporated into the enamel pellicle and plaque may explain this phenomena (21).

In the light of these reflections, we aimed to assess the effect of Nd:YAG laser irradiation following CPP-ACP application on enamel concentration of calcium and phosphate. Also, we tested if enamel microhardness varies with the combined use of Nd:YAG laser and CPP-ACP compared to CPP-ACP or laser treatments alone. We expected that combination of Nd:YAG laser and CPP-ACP in comparison with any of these treatments alone induces a remarkable increase in calcium and phosphate content of artificial early enamel caries and, thereby, significant improvement in enamel microhardness.

## Material and Methods

-Sample size 

Eighty enamel samples in four groups were used for the purpose of this study. A random allocation list generated using randomization software (RandList version 1.2; DatIng GmbH, Tübingen, Deutschlan) was used to allocate the specimens to each group including 20 samples.

• Group A: Distilled and deionized water (negative control).

• Group B: Nd:YAG laser (positive control).

• Group C: CPP-ACP crème (positive control).

• Group D: CPP-ACP crème and Nd:YAG laser (test group).

According to a pilot study, the mean value for total weight percentage of calcium in the groups A, B, C, and D were 5.18 ± 2.21, 8.52 ± 2.84, 11.90 ± 6.20, and 14.85 ± 3.94, respectively. Considering α = 0.05, power = 0.8 and difference = 1.75 in the total weight percentage of calcium, 18 samples were estimated for each group (total of 72 samples). The sample size was increased to 80 to improve the validity of study.

-Specimen preparation

Exfoliated human upper and lower primary teeth were used for this study. The roots of the teeth were removed and the teeth were thoroughly cleaned with a rubber cup/pumice prophylaxis. The teeth were stored at 4°C in the deionized water containing 0.05% thymol after rinsing under tap water. Teeth with defects, erosions, microcracks as well as visible stains identified using a magnifying lens were excluded. To mount the samples, custom-made plastic cylindrical moulds were prepared, and the self-cured acrylic resin was poured in the moulds. Each enamel block was embedded in the self-cured acrylic resin with the buccal surface exposed.

The labial surfaces were ground flat and polished using a Sof-Lex polishing and finishing disks (3M/ESPE, St Paul, MN, USA) in progressively finer grits (coarse, medium, fine and superfine) with low-speed handpiece. An acid-resistant nail varnish was applied around the exposed enamel surface, leaving a window (2×2 mm) of the enamel exposed at the center (22).

-Lesion formation

The sectioned specimens of the enamel blocks were immersed in the demineralized solution containing 2.2 mM Ca(NO3)2, 2.2 mM phosphate as KH2PO4, 0.1 ppm NaF, and 50 mM acetic acid at 37°C for 48 h 22.

-Remineralization

Artificial saliva was used as a remineralizing solution (pH = 7.0) according to a previously described method (22).

In the control group, no treatment was given to the enamel surfaces of the specimens, and the teeth were kept in the deionized water for 5 min (22).

In the Nd:YAG group, the specimens surface were treated with the Nd:YAG laser (LAMBDA Sicentifica, S. r. l, Vicenza, Italy) emitting at 1.064 μm wavelength. The laser beam was delivered in the non-contact mode, perpendicular to the specimen surface through a quartz fiber with a diameter of 200 μm and irradiation time of 15 s: 100 mJ energy with 125 J/cm2, 10 Hz repetition rate and 1 W power (23).

In the CPP-ACP group, a thin layer of the CPP-ACP crème was applied, using a microbrush, left intact for 5 min (22).

In the CPP-ACP crème combined with Nd:YAG laser group, a thin layer of the CPP-ACP crème was applied using a microbrush, and then, the specimens surfaces were treated with the Nd:YAG laser for 15 s and left undisturbed for 5 min.

All the specimens were washed with the deionized water after the remineralization process, and were left in the artificial saliva during the remaining time (approximately 20 h ⁄ day). The specimens underwent the remineralization process with CPP-ACP crème twice a day (08:00 am, 3:00 pm) for 30 days (22).

Then, enamel surface microhardness (SMH) of each specimen was measured. To this end, an enamel SMH tester (UHL VMH,Germany) with a Vickers diamond indenter was used with a 30 g load for 15 s. Five indentations were made on the surface of each specimens to obtain a mean Vickers hardness number (VHN) (23).

The mineral content (calcium and phosphate) of all samples were assessed using scanning electron microscopy (SEM) (MIRA3, FEG-SEM, TESCAN, Czech Republic) and energy dispersive X-ray (EDX) tests. EDX analysis was performed for the samples with a Norvar EDS X-ray detector capable of detecting elements with atomic weights as low as that of Beryllium (Be) (22). Then, the two samples of each group were coated with a 25 nm layer of gold by desk sputter coater and placed on the storage of a SEM (MIRA3, FEG-SEM, TESCAN, Czech Republic) with an accelerating voltage of 30 keV and beam current of 1.000 nA in order to photographing and observing morphological changes (23).

-Statistical analysis

The data were analyzed using SPSS 17/win software. Descriptive statistics including the mean, standard error of mean, and minimum and maximum values were calculated for all variables using Kruskal-Wallis test and pair wise comparisons of the study groups were performed using the Mann-Whitney U test. A significant level of α = 0.05 was set for comparison between the groups.

## Results

-Total weight percentage of calcium, phosphate and enamel surface micro hardness

The mean enamel VHN and total weight percentage of calcium and phosphate, resulting from the EDX analysis of enamel surface in treatment groups are given in  Table 1.

**Table 1:**
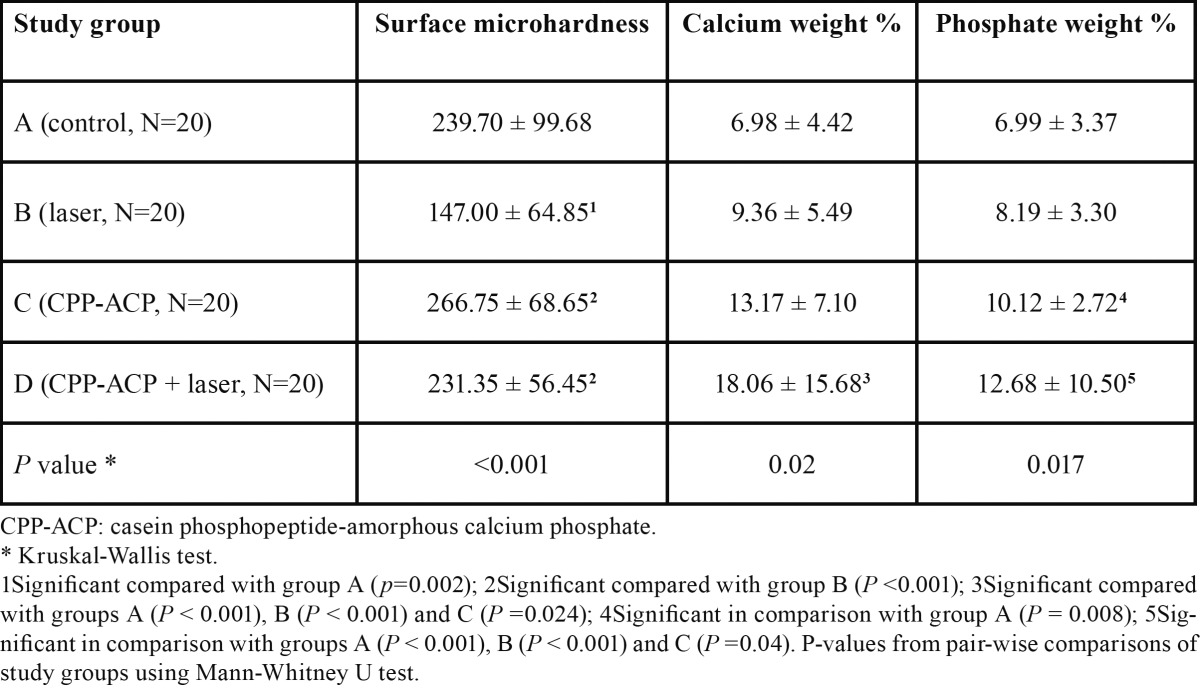
Comparison of mean values (mean ± SD) of surface microhardness, calcium weight percentage, and phosphate weight percentage in the studied groups.

Mean total weight percentage of calcium was higher in group D compared to other groups ( Table 1). Also mean total weight percentage of phosphate was higher in group D compared to other groups. The difference in the mean values were statistically significant between groups A and D and also between groups A and C, while the difference was not significant between groups A and B ( Table 1).

The highest mean VHN was recorded in group C ( Table 1). The results showed a statistically significant difference in the mean values of VHN between groups A and B, while the difference was not significant between groups A and other groups. Mean VHN in groups D and C were significantly higher than that in group B; meanwhile, this difference was not significant between groups D and C the p values are listed in  Table 1.

-SEM micrograph findings

**Figure 1:**
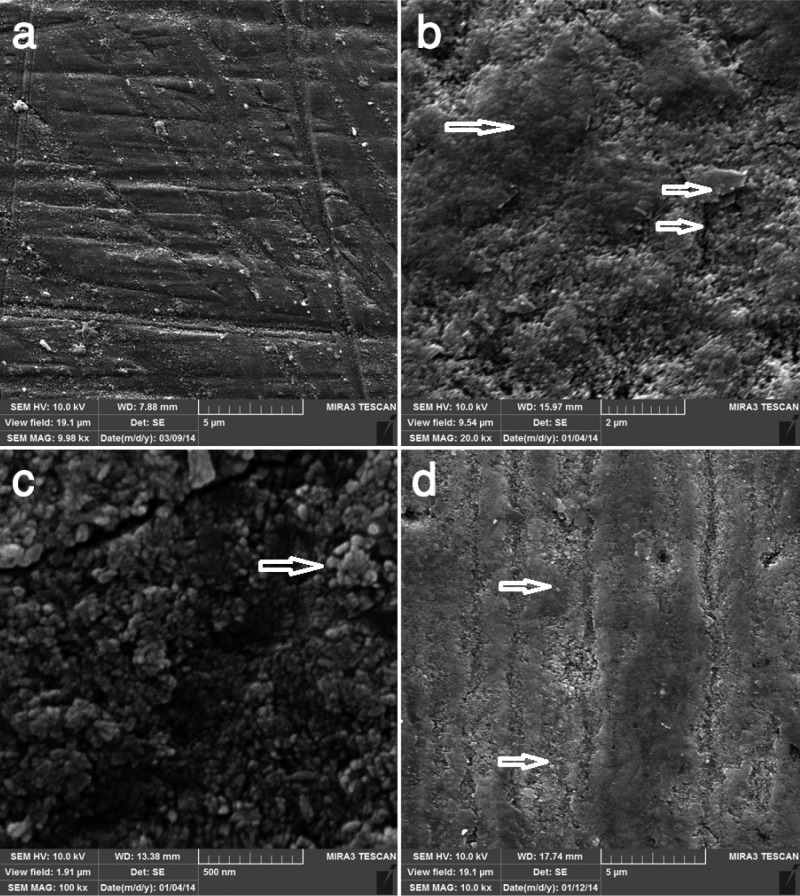
a) SEM image of control group showing enamel surface. b) SEM image of enamel surface irradiated with Nd:YAG laser showing melted enamel (large arrow) and fine cracks (small arrow). c) SEM image of enamel surface treated with CPP-ACP showing granular particles and amorphous crystals (arrow). d) SEM image of enamel surface treated with combined application of CPP-ACP and laser showing smooth and more homogeneous lazed surface (large arrow) with a few cracks coating by granular and globular particles and amorphous homogenous crystals (small arrow).

Figure 1a shows a sound enamel surface from the negative control group demonstrating relatively smooth area with frequent enamel prism ends present on the surface. The surface presented shallow depressions and fine porosities within these depressions. The enamel surfaces were devoid of any surface deposits.

The SEM image of enamel irradiated with Nd:YAG laser (group B) showed an irregular roughened surface (Fig. 1b). In this group, typical melting appearance with occasional areas of fine surface cracking, discontinuities, coalescence of globules and holes could be observed. The surface was peppered with cavitations and craters leading to an irregular undulated surface. The surface did not show any of the usual enamel prism ends seen in the control group. Numerous cracked surface coatings covered almost the entire surface.

In the SEM image of the enamel surface treated with CPP-ACP (group C), numerous granular particles and amorphous crystals were arranged on the enamel surface, those crystals seemed to be homogeneous, and there was no obvious intercrystalline space (Fig. 1c).

In the CPP-ACP plus laser samples (group D), a relatively smooth, more homogeneous surface compared to those in the groups treated with laser and CPP-ACP alone were noted (Fig. 1d). Fewer cracks were observed on the glazed enamel surface, probably due to the application of CPP-ACP before laser irradiation, consistent with the numerous occurrences of granular and globular particles and amorphous homogenous crystals arranged on the enamel surface.

## Discussion

The combination of laser and fluoride treatment had previously shown to enhance the resistance of enamel by increasing the uptake of fluoride by enamel and consequently reducing the acid dissolution rate of enamel (20,24). In this study, we aimed at reconstructing this model using laser irradiation followed by CPP-ACP treatment.

Based on the supportive findings of the present and previous studies regarding the tested hypothesis, three primary mechanisms involved in the cariostatic effect of laser irradiation in conjunction with CPP-ACP treatment can be explained.

-Increased calcium and phosphate deposition in enamel subsurface

In the present research, the highest mean weight percentage of calcium and phosphate was obtained for the CPP-ACP and laser treated subjects followed by CPP-ACP alone, lasing alone and negative control subjects. Similarly, previous studies have demonstrated that application of the CPP-ACP alone could enhance the subsurface calcium and phosphate concentrations (25) compared to calcium and phosphate in the saliva and preserves them in close proximity to the enamel lesion, thereby decreasing demineralization and enhancing remineralization of enamel lesions (26). In addition, a significant increase in the mean percentages of calcium and phosphate in CPP-ACP plus laser group compared to that in CPP-ACP group could be attributed to the pattern typically described as mechanisms involved in reduction of enamel permeability (11,27) and increase in fluoride uptake on the enamel surface treated by laser (28). During the melting and re-solidifying processes caused by laser treatment, many partially coalescent globular granules form on the enamel surface and fluoride ions are provided with an opportunity to penetrate into the microspaces between these granules (4). In a similar pathway, use of CPP-ACP increases the subsurface concentration of calcium and phosphate (25). Thereafter, laser irradiation can saturate calcium and phosphate on the enamel surface which can be easily up taken by the enamel surface and trapped into the spaces between the granules. Similar phenomenon has also been described by Westerman *et al.* (29) who have demonstrated an increase in fluoride, calcium, and phosphate uptake from exogenous sources as one of the irradiation-induced caries resistance mechanism. Thus, it seems to be logical to assume a similar mechanism of effect to the observed increase in up taking and retaining calcium and phosphate in CPP-ACP plus laser group compared to other groups and a significant increase in enamel microhardness of CPP-ACP plus laser group compare to laser group. This result was also confirmed by the SEM images which showed melted enamel and coalescence of globules with apparent irregularities and holes in the outer layer of the enamel of laser group (Fig. 1b). The findings from these micrographs are in agreement with the findings of Tagomori & Iwase and Souza *et al.* (4,30). In addition, SEM images of CPP-ACP group revealed that numerous granular particles and amorphous crystals were arranged on the enamel surface, in line with the findings of Zhang *et al.* (22). These findings are probably related to deposition of calcium and phosphate on enamel surface and subsurface, and thereby, formation of new crystals and granules. Therefore, remineralization of subsurface lesions in CPP-ACP treated subjects may occur as a result of the deposition of the mineral ions and formation of hydroxyapatite crystals and have an important role in enhancing SMH values in this group.

Interestingly, the SEM images of CPP-ACP and laser treated subjects showed a smooth and more homogeneous glazed surface with a fewer number of cracks coated with globular and granular deposits and amorphous crystals (Fig. 1d). According to previous studies, laser treatment could enhance fluoride uptake and the CPP-ACP treatment could enhance mineral content of enamel surface (4,25). It could be concluded that, when laser irradiation is combined with the application of CPP-ACP, fluoride ions come into contact with free calcium and phosphate ions, and as a consequence, new hydroxyapatite and fluorapatite crystals form on the enamel surface, coating the cracks to form a smoother surface with higher SMH compared to laser treated group.

-Re-crystallizing hydroxyapatite crystals

Comparing with the result of earlier studies performed with this model on fluoride, it can be concluded that the use of Nd:YAG laser and the melting of the enamel (Fig. 1b) recrystallizes forming hydroxyapatite crystals to those larger than the initial ones and better retained on the enamel surface (7,29). It seems when Nd:YAG laser is used alone, the main mechanism is the enhanced acid resistance through recrystalization and there is minimal effect on the enamel subsurface calcium and phosphate concentration. In laser treatment following CPP-ACP application, however, the main mechanism could be related to the enhancement of enamel subsurface calcium and phosphate content as a result of CPP-ACP application and fluoride uptake by laser irradiation, resulting in localization of ACFP at the tooth surface by the CPP, which colocalizes calcium, phosphate and the fluoride as bioavailable ions to form fluorapatite. Formation of new amorphous crystals and globular deposits along the fine cracks of melted coating enamel surface observed in SEM images of subjects treated with combination of CPP-ACP and laser (Fig. 1d) is supportive of the described mechanism. While the SEM micrographs of laser group showed only melted surface with fine cracks without crystals (Fig. 1b), the SEM images of CPP-ACP group revealed granular particles on enamel surface that were less homogeneous compared to the specimens of CPP-ACP plus laser group (Fig. 1c).

In addition, based on our results, recrystallizing of enamel crystals formed by laser irradiation could retain calcium and phosphate ions better than non-irradiated enamel, probably due to penetration of these ions into the spaces between globular granules formed by recrystalization of enamel crystals, and can explain the significantly higher SMH in CPP-ACP plus laser group compared to group B.

-Purification of enamel hydroxyapatite

Comparison of SMH between groups revealed an essential theme underlying observed structural differences. The mean VHN in group CPP-ACP plus laser was significantly higher than that in group B. Although the difference did not reach statistical significance, the mean VHN in group CPP-ACP plus laser was lower than those in groups control and CPP-ACP. Additionally and more importantly, this value in group B was significantly lower than the other groups. Mean VHN was found to be significantly higher in the untreated control group compared to that of laser group. This decrease in enamel SMH in group B could be attributed to the effect of laser radiation in producing fine irregularities and cracks in enamel surface, favoring brittleness of the enamel, as seen in the SEM images of laser group that revealed melting and fusion with the formation of cracks and holes on the enamel surface (Fig. 1b). The cracks of enamel surface were not seen in the SEM micrographs of CPP-ACP group (Fig. 1c) and were coated with amorphous crystals and globular deposits in the CPP-ACP plus laser group (Fig. 1d). These cracks act as starting point for acid attack and make enamel brittle, leading to decreased SMH. However, previous studies have been inconclusive mainly because the probable diversity of methodologies and the differences in the applied substrates, i.e., primary enamel and lasing source parameters. Marquez *et al.* (31) using a 5 kHz Q-switched Nd:YAG noted an increase in SMH due to the fusion of enamel surface. However, Tagomori & Iwase, Kuramoto *et al*, and Jennet *et al.* showed a decrease in the SMH values of the lased enamel (4,32,33). Conversely and in line with our result, Kuramoto’s study revealed that the samples lased with low energy (3-21 j) showed similar SMH value, while those subjected to the higher energy (30-100 j) showed lower values than control subjects (33). This result was attributed to the fact that higher energy produces bigger cracks and consequently brittle enamel leading to decreased SMH.

Further, it could be concluded that application of CPP-ACP in groups C and D in the present study resulted in a marked increase in subsurface calcium and phosphate and co-localization of these ions with fluoride as bioavailable ions to form fluorapatite and thereby enhancing mean enamel SMH. In addition, apatite formed in an environment containing more calcium and phosphate is expected to contain less CO2 and less Mg by replacing them with calcium and phosphate, making enamel more acid resistant (34) and increasing its SMH levels. This finding is comparable to the results of Zhang Q *et al.* (22) study.

On the other hand, it is plausible to postulate that laser irradiation following CPP-ACP application enhances subsurface calcium and phosphate, and consequently fluoride ions come into contact with free calcium and phosphate ions. The localization of ACFP at the enamel surface could reduce or eliminate negative effects of laser irradiation. The combined application of CPP-ACP and laser irradiation could create a reservoir for calcium and phosphate in partially coalescent globular granules that are produced by the process of melting and the subsequent resolidification of enamel crystals. Consequently, calcium and phosphate can easily penetrate into the spaces between the granules formed by laser irradiation.

The findings of the present study may lead to a paradigm shift in the remineralization treatment of early enamel lesions using laser irradiation following CPP-ACP treatment. However, such conclusive inference should be weighed against some limitations of the study. Regarding the fact that this *in vitro* study used a small sample size in each group, it would be beneficial to perform similar *in vitro* study using more samples in all groups that might increase the level of significant differences among and between the groups. Including additional groups, such as CPP-ACP containing fluoride and different laser types and energies allow further assessment of the interaction of these products.
